# Characterization of the HIV-1 RNA associated proteome identifies Matrin 3 as a nuclear cofactor of Rev function

**DOI:** 10.1186/1742-4690-8-60

**Published:** 2011-07-20

**Authors:** Anna Kula, Jessica Guerra, Anna Knezevich, Danijela Kleva, Michael P Myers, Alessandro Marcello

**Affiliations:** 1 Laboratory of Molecular Virology, International Centre for Genetic Engineering and Biotechnology (ICGEB), Padriciano, 99, 34012 Trieste, Italy; 2 Laboratory of Protein Networks, International Centre for Genetic Engineering and Biotechnology (ICGEB), Padriciano, 99, 34012 Trieste, Italy; 3 Department of Microbiology and Molecular Medicine, University of Geneva, Geneva, 1211, Switzerland

## Abstract

**Background:**

Central to the fully competent replication cycle of the human immunodeficiency virus type 1 (HIV-1) is the nuclear export of unspliced and partially spliced RNAs mediated by the Rev posttranscriptional activator and the Rev response element (RRE).

**Results:**

Here, we introduce a novel method to explore the proteome associated with the nuclear HIV-1 RNAs. At the core of the method is the generation of cell lines harboring an integrated provirus carrying RNA binding sites for the MS2 bacteriophage protein. Flag-tagged MS2 is then used for affinity purification of the viral RNA. By this approach we found that the viral RNA is associated with the host nuclear matrix component MATR3 (Matrin 3) and that its modulation affected Rev activity. Knockdown of MATR3 suppressed Rev/RRE function in the export of unspliced HIV-1 RNAs. However, MATR3 was able to associate with Rev only through the presence of RRE-containing viral RNA.

**Conclusions:**

In this work, we exploited a novel proteomic method to identify MATR3 as a cellular cofactor of Rev activity. MATR3 binds viral RNA and is required for the Rev/RRE mediated nuclear export of unspliced HIV-1 RNAs.

## Introduction

Viruses have evolved to optimize their replication potential in the host cell. For this purpose, viruses take advantage of the molecular strategies of the infected host and, therefore, represent invaluable tools to identify novel cellular mechanisms that modulate gene expression [1].

The primary viral transcription product is utilized in unspliced and alternatively spliced forms to direct the synthesis of all human immunodeficiency virus (HIV-1) proteins. Although nuclear export of pre-mRNA is restricted in mammalian cells, HIV-1 has evolved the viral Rev protein to overcome this restriction for viral transcripts [2,3], recently reviewed in [4]. Rev promotes the export of unspliced and partially spliced RNAs from the nucleus through the association with an RNA element called the Rev response element (RRE) that is present in the *env *gene [5-7]. In the cytoplasm, the RRE-containing HIV-1 transcripts serve as templates for the expression of viral structural proteins, and the full-length unspliced forms serve as genomic RNAs that are packaged into viral particles. In order to fulfill its function, Rev requires the assistance of several cellular cofactors (reviewed in [8]). Rev interacts with a nucleocytoplasmic transport receptor, Exportin 1 (CRM1), to facilitate the export of viral pre-mRNAs [9]. Rev also engages the activity of cellular RNA helicases [10] and capping enzymes [11] that are required for the correct nuclear export of Rev interacting viral RNAs.

The nucleus is a complex organelle where chromosomes occupy discrete territories and specific functions are carried out in sub-nuclear compartments [12-15]. Transcription, for example, has been proposed to occur in 'factories' where genes and the RNA polymerase complex transiently assemble [16,17]. Once integrated, the HIV-1 provirus behaves like a cellular gene, occupying a specific sub-nuclear position and takes advantage of the cellular machinery for transcription and pre-mRNA processing [18-21]. Control of HIV-1 gene expression is critical for the establishment of post-integrative latency and the maintenance of a reservoir of infected cells during antiretroviral therapy [22]. Beyond transcriptional control, processing of the RNA may also concur in the establishment of a latent phenotype [23].

The spatial positioning of chromatin within the nucleus is maintained by a scaffold of filamentous proteins generally known as the nuclear matrix [24]. Although the exact function of the nuclear matrix is still debated [25], several of its components have been implicated in nuclear processes that include DNA replication, repair, transcription, RNA processing and transport [26-28]. Matrin3 (MATR3) is a highly conserved component of the nuclear matrix [29-31]. MATR3 is a 125 kDa protein that contains a bipartite nuclear localization signal (NLS), two zinc finger domains, and two canonical RNA recognition motifs (RRM) [32]. Little is known about the function of MATR3. A missense mutation in the MATR3 gene has been linked to a type of progressive autosomal-dominant myopathy [33]. MATR3, together with the polypyrimidine tract-binding protein associated splicing factor (PSF) and p54^nrb^, has been implicated in the retention of hyperedited RNA [34]. Recently, MATR3 has also been involved in the DNA damage response [35]. Hence, MATR3 may be at the crossroad of several nuclear processes, serving as a platform for the dynamic assembly of functional zones of chromatin in the cell nucleus in a so-called 'functional neighborhood' [36].

In the present work, we developed a novel proteomic approach for the identification of host factors involved in nuclear steps of HIV-1 RNA metabolism. In our proteomic screen, we identified MATR3, and we provide evidence that it binds viral RNA and is required for Rev- activity.

## Results

### Generation and characterization of cell lines expressing tagged HIV-1 RNAs

The MS2 phage coat protein is a well-described tool for RNA tagging [37]. Modified MS2 homodimers bind with high affinity to a short RNA stem loop that can be engineered in multimers in the RNA of interest for various purposes. On one hand, MS2 fused to the green fluorescent protein (GFP) has been used to visualize mRNAs in living cells allowing for the kinetic analysis of mRNA biogenesis and trafficking [38-40]. Alternatively, MS2 fused to the maltose binding protein (MBP) has been used to purify the spliceosome by affinity chromatography of cellular extracts [41]. Recently, to visualize and analyze the biogenesis of HIV-1 mRNA, we inserted twenty-four MS2 binding sites in the 3'UTR of an HIV vector and demonstrated that this system fully recapitulates early steps of HIV-1 transcription [42,43].

**Figure 1:**
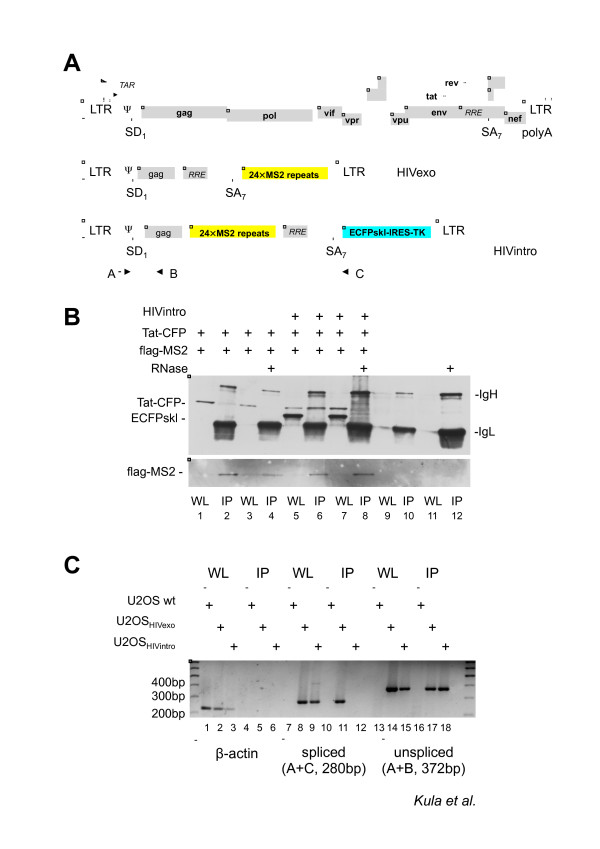
**Detection and identification of HIV-1 RNA associated factors**. A) Description of the HIV-1 constructs. Above an outline of the full-length viral genome, below the two constructs used in this work: HIVexo (carrying the MS2 binding sites after the SA7 splice site) and HIVintro (carrying the MS2 repeats in the intron). Black arrows indicate the RT-PCR primers listed in Table 2. The scheme is not drawn to scale. B) Pulldown of HIV-1 RNA and associated Tat. 293T cells expressing the indicated constructs were lysed and immunoprecipitated with anti-flag beads. Immunoblots with anti-GFP antibodies show Tat-CFP (lanes 1, 3, 5 7) and ECFPskl (lanes 5 and 7) expressed by the HIVintro construct. Tat could be immunoprecipitated only when the HIV-1 RNA is present and the association is disrupted by RNase treatment (compare lanes 6 and 8). IgH and IgL are the heavy and light chains of the immunoglobulins used in the immunoprecipitation. IP and WL stand for immunoprecipitation and whole cell's lysate, respectively. C) MS2-dependent pulldown of specific HIV-1 RNAs. U2OS clones and U2OS wt cells expressing Tat-CFP and flag-MS2nls were lysed and immunoprecipitated with anti-flag beads. RNA was extracted from immunoprecipitations and the RNA reverse-transcribed and PCR amplified with primers for β-actin mRNA (lanes 1-6), as well as with primers that differentiate spliced (lanes 7-12) and unspliced (lanes 13-18) forms of the HIV-1 RNAs which are outlined in Figure 1A.

In this work, we aimed to develop an MS2-based approach to identify novel host factors associated with HIV-1 RNA. To this end we took advantage of two HIV-1 derived vectors called HIV_Exo_24 × MS2 (HIVexo) and HIV_Intro_24 × MS2 (HIVintro), described earlier [42-45], which carry the MS2 tag either in the exonic or in the intronic part of the viral sequence, respectively (Figure 1A andAdditional File 1). These HIV-1 reporter vectors contain the *cis *acting sequences required for viral gene expression and downstream steps in replication: the 5' LTR, the Tat responsive region TAR, the major splice donor (SD1), the packaging signal ψ, a portion of the *gag *gene, the Rev responsive region RRE, the splice acceptor SA7 flanked by its regulatory sequences (ESE and ESS3), and the 3' LTR that drives 3'-end formation (Figure 1A). The HIVintro vector carries additionally the reporter gene coding for the cyan fluorescent protein fused with peroxisome localization signal (ECFPskl). Moreover, placement of the 24xMS2 tag inside the intron of the HIVintro vector increases the probability of purifying proteins involved in early nuclear steps of HIV-1 RNA processing [44]. To demonstrate that it was feasible to pull-down proteins associated with viral RNA *via *flag-tagged MS2, we transfected 293T cells with HIVintro, together with a construct expressing the Tat *trans*-activator fused to CFP and a construct expressing a flag-tagged MS2nls. Total cell extracts were immunoprecipitated with anti-flag antibodies and blotted against GFP or flag. As shown in Figure 1B, Tat-mediated viral expression is indicated by the presence of reporter CFPskl in the lysates (lanes 5 and 7). Importantly, Tat-CFP is immunoprecipitated when pHIVintro is present, but the interaction is lost in the presence of RNase (compare lane 6 and 8) demonstrating that HIV-1 RNAs carrying both the TAR and the MS2 repeats are required to pull down Tat-CFP.

Next, two U2OS cell lines carrying stable arrays of either HIVexo or HIVintro were selected that show robust *trans*-activation by Tat and other stimuli known to induce transcription of integrated HIV-1 [42,43]. To demonstrate that our strategy was able to distinguish between the unspliced and spliced viral RNAs in the pull-down, U2OS HIVintro and U2OS HIVexo cells were transfected with plasmids expressing Tat-CFP and flag-MS2nls. Cell lysates were immunoprecipitated with anti-flag antibodies, extensively washed and used as templates for RT-PCR using primers that are able to distinguish unspliced (A+B, 372 bp) and spliced (A+C, 280 bp) RNAs. As shown in Figure 1C, only the spliced RNA of HIVexo (lane 11), but not of HIVintro (lane 12), was immunoprecipitated, whereas both unspliced RNAs could be detected (lanes 17, 18). The absence of the spliced product in the pull-down from HIVintro is explained by the loss of the MS2 tag after splicing and demonstrates the specificity of the MS2-based RNA affinity purification. Moreover, detection of unspliced HIV RNA in both IPs reinforces the notion that a certain proportion of this product is maintained during transcription of HIV-1. All together these observations show that the MS2-based strategy can be successfully used for the purification of factors interacting with viral transcripts.

### Identification of proteins associated with HIV-1 RNA

**Figure 2:**
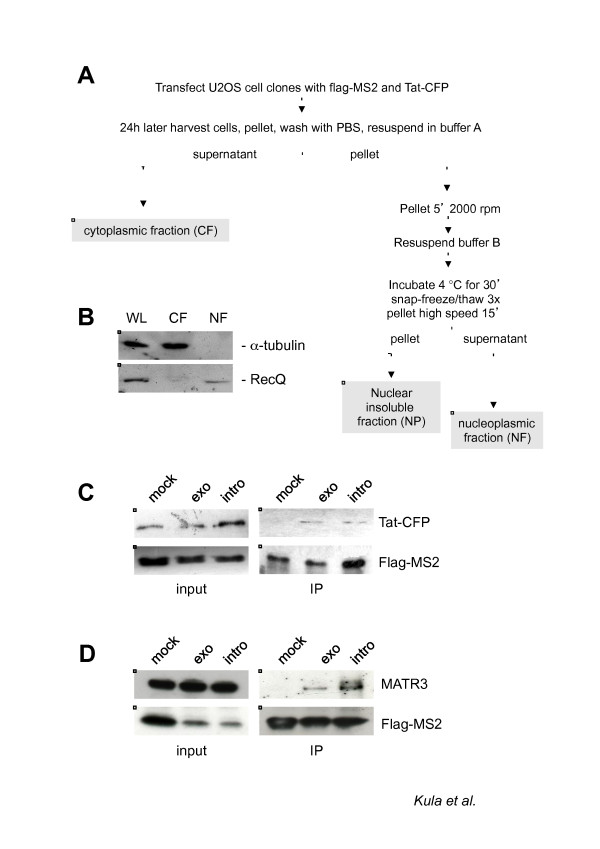
**Immunoprecipitation of HIV-1 RNA from nucleoplasmic fractions**. A) Biochemical fractionation for the proteomic analysis. Nuclear extraction scheme showing the various phases of the protocol used to produce the nucleoplasmic fraction. B) Control of nuclear extraction in U2OS cells. The fractions obtained by the protocol outlined in Figure 2A were loaded on a gel for immunoblotting against α-tubulin (upper panel) that shows up only in the cytoplasmic fraction (CF) and against the nuclear protein RecQ (bottom panel) that was present only in the nucleoplasmic fraction (NF). C) Control of HIV-1 RNA associated factor Tat in the NF. Nuclear extracts from U2OS cells (mock), U2OS HIV_Exo_24 × MS2 (exo) or U2OS HIV_Intro_24 × MS2 (intro) were immunoprecipitated for HIV-1 RNA as described above, loaded on SDS-PAGE and blotted against GFP to detect the RNA-bound Tat-CFP protein (IP). Immunoblots for the nuclear extracts against GFP and flag-MS2nls (input) are shown. D) Pulldown of HIV-1 RNA and endogenous MATR3. Whole cell extracts from U2OS cells (mock), U2OS HIV_Exo_24 × MS2 (exo) or U2OS HIV_Intro_24 × MS2 (intro) were immunoprecipitated for HIV-1 RNA as described above, loaded on SDS-PAGE and blotted against MATR3 to detect the RNA-bound endogenous protein (IP). Immunoblots for the whole cell extracts against MATR3 and flag-MS2nls (input) are shown.

As we described above, we used the MS2 tagging for the purpose of HIV-1 RNA affinity purification. Next, to identify nuclear factors associated with viral RNA, we proceeded as follows: U2OS HIVexo and U2OS HIVintro stable cell lines together with wild type U2OS were transfected with vectors expressing Tat-CFP and flag-MS2nls proteins. Since we were interested in the identification of factors involved in nuclear HIV-1 RNA metabolism, we subjected the cells to biochemical fractionation for the extraction of the nucleoplasmic fraction (NF) (Figure 2A). Indeed, the procedure resulted in clean preparation of NF as controlled by immunoblotting with nuclear (tubulin) and cytoplasmic (RecQ) markers as shown in Figure 2B. The nuclear fraction was further subjected to flag-immunoprecipitation. IPs were extensively washed in the presence of nonspecific competitors as described in Materials and Methods, and the specificity of pulldown was assessed by immunoblotting as shown in Figure 2C. Lastly, IPs were subjected to mass spectrometry analysis as described in details in Materials and Methods. We were interested in proteins that associated with both HIVexo and HIVintro RNAs because they represent hits obtained from two totally independent procedures. The combined results of two immunoprecipitations led to the identification of 32 proteins that were specific for the stable cell lines carrying the virus (Table 1). Indeed, most of the identified proteins have been characterized in RNA binding and/or regulation. Proteins such as BAT1, FUS and hnRNPs have been already found in large-scale proteomic analysis of the human spliceosome [46,47]. BAT2 and CAPRIN1 were shown to associate with pre-mRNA, although their role in pre-mRNA processing is yet to be demonstrated [48,49]. Interestingly, many of the identified proteins have been already shown to be involved in various steps of HIV-1 RNA metabolism. DBPA and RPL3 were shown to interact with the TAR while ILF3 interacts with both - the TAR and the RRE [50-52]. DDX3X, SFPQ and Upf1 were shown to regulate Rev-dependent unspliced and partially spliced viral transcripts while PTB was shown to regulate Rev-independent, multiply spliced HIV-1 RNA [10,23,53,54]. MOV10 belongs to a family of Upf1-like RNA helicases, and it has been shown to inhibit viral replication at multiple stages although its activity on viral RNA is yet to be discovered [55,56]. Interestingly, in both screens we identified the nuclear matrix protein MATR3 as a strong candidate according to the number of non-redundant peptides sequenced (the log(e) score was -44.4 for U2OS HIVintro and -38.2 for U2OS HIVexo). MATR3 is of particular interest because very little is known about its nuclear function, and it has never been described in the context of HIV-1 replication. Although MATR3 contains two canonical RNA recognition motifs (RRM), its RNA target is unknown. Intriguingly, MATR3 was shown to interact with the SFPQ/p54^nrb ^complex which triggers the nuclear retention of A to I hyperedited RNA [34]. Therefore, we were stimulated to further investigate the possible MATR3 interaction with HIV-1 RNA.

**Table 1:** Proteins identified by mass spectrometry.

Gene ID	Proposed function(s)	**Entrez n. & Ref**.
**Pre mRNA/mRNA binding proteins **[41,46,47]		

BAT1	RNA helicase (UAP56) also involved in RNA export	7919

FUS	Oncogene TLS (Translocated in liposarcoma protein) is a multifunctional RNA-binding protein factor	2521

HNRPA3	heterogeneous nuclear ribonucleoprotein	220988

HNRPDP	heterogeneous nuclear ribonucleoprotein (hnRNP D0)	8252

HNRPF	heterogeneous nuclear ribonucleoprotein	3185

HNRPM	heterogeneous nuclear ribonucleoprotein	4670

HNRPR	heterogeneous nuclear ribonucleoprotein	10236

VIM	Vimentin, structural constituent of cytoskeleton	7431

**Other pre-mRNA/mRNA associated proteins**		

BAT2	May play a role in the regulation of pre-mRNA splicing	7916 [48]

C14orf166	hCLE/CGI-99 is a mRNA transcription modulator	51637[77]

CAPRIN1	GPI-anchored membrane protein 1/p137 associates with human pre-mRNA cleavage factor II_m_	4076 [49,78]

GAPDH	Glyceraldehyde-3-phosphate dehydrogenase, also shown to bind ssDNA/RNA and to have a role in RNAPII histone genes activation	2597 [79,80]

**Involved in HIV RNA binding/regulation**		

DBPA	YB-1 interacts with TAR and Tat (*)	8531 [50]

DDX3X	Involved in Rev-mediated non-terminally spliced RNA export (*)	1654 [10]

EEF1A1	Involved in RNA-dependent binding of Gag	1915 [81]

ILF3	NF90 binds HIV-1 TAR and RRE (*)	3609 [51,52]

MOV10	RNA helicase that inhibits HIV-1 replication	4343 [55,56]

PTBP1	PTB has been involved in nuclear retention of multi-spliced HIV mRNAs in the nucleus of resting T cells (*)	5725 [82]

TUBA1B	HIV-1 Tat binds tubulin (*)	10376 [83]

RPL3	Also described as HIV-1 TAR RNA-binding protein B (TARBP-b)	6122 [84]

SFPQ	PSF is involved in Rev-mediated export of HIV-1 RNA (*)	6421 [53]

UPF1	Upframeshif protein 1 RNA helicase. Part of a post-splicing multiprotein complex.	5976 [54,85]

**Other**		

CFL1	It is the major component of nuclear and cytoplasmic actin rods.	1072

EIF4A1	ATP-dependent RNA helicase; eIF4F complex subunit involved in cap recognition and is required for mRNA binding to ribosome.	1973

HIST1H1A	histone 1, H1a	3024

H1FX	histone 1 family, H1 member X	8971

PRKDC	DNA-dependent protein kinase (DNA-PKcs) involved in dsDNA break repair	5591

RIF1	Associated with aberrant telomers and dsDNA breaks	55183

SCYL2	Putative kinase in yeast	55681

SPIN1	Spindlin 1 belongs to the SPIN/STSY family	10927

(*) also identified as pre-mRNA/mRNA binding proteins [41,46,47].

To confirm that MATR3 specifically co-immunoprecipitates with viral RNA, we transfected U2OS HIVexo and U2OS HIVintro stable cell lines and wild type U2OS with flag-MS2nls and Tat. Cells were lysed, and the resulting cell extract was subjected to immunoprecipitation with anti-flag antibodies. Resulting pulldowns were immunoblotted with MATR3 and flag antibodies. As shown in Figure 2D, MATR3 is detected on flag-MS2 pulldown only in cells expressing the HIV vectors, both HIVexo and HIVintro, and not in mock cells confirming that MATR3 interacts with HIV-1 RNA.

Our preliminary observations suggest that MATR3 is a novel HIV RNA-binding factor. Therefore, we decided to further investigate the functional meaning of this interaction.

### MATR3 is required for Rev activity

**Figure 3:**
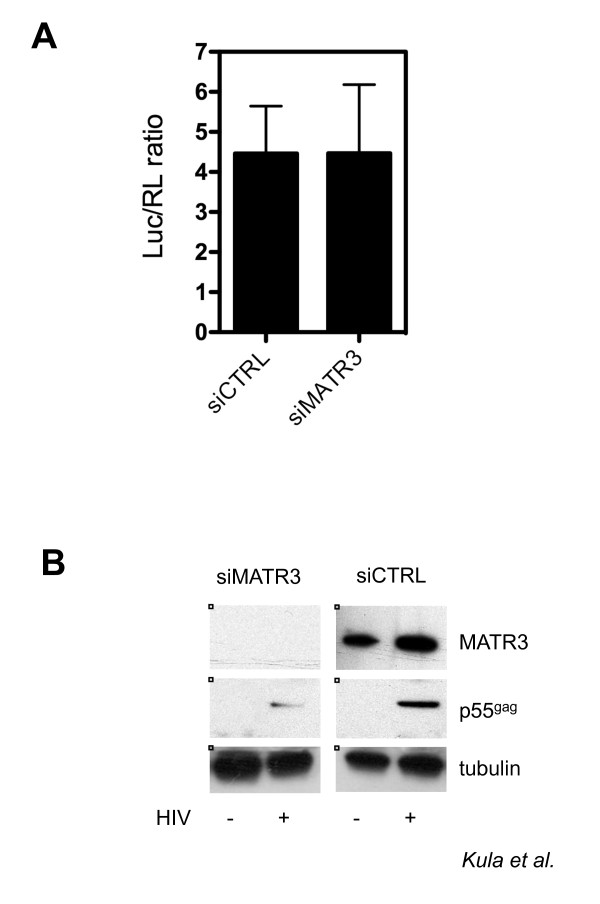
**MATR3 is a post-transcriptional cofactor of HIV-1**. A) MATR3 knockdown does not affect the luciferase activity. HeLa cells were transfected with the indicated siRNAs. After 48 hours siRNA-treated cells were transfected with the pNL4.3R-E-luc HIV-1 molecular clone and with pCMV-Renilla and harvested 24 hours later for luciferase assays. Relative Luc/RL expression was normalized to protein levels measured by Bradford assay. The results of three independent experiments are shown ± SD. B) MATR3 knockdown leads to decrease of the Gag expression from pNL4.3R-E-luc HIV-1 molecular clone. HeLa cells were transfected with the siRNA targeting MATR3 (siMATR3) or with a control siRNA (siCTRL). After 48 hours siRNA-treated cells were transfected with pNL4.3R-E-luc and harvested 24 hours later for immunobloting. Tubulin is the protein loading control.

To investigate the functional role of MATR3 in HIV-1 replication, we measured the effect of RNAi-mediated knockdown on a full-length HIV-1 molecular clone carrying the luciferase reporter gene in *nef *(pNL4.3R-E-luc). As shown in Figure 3A, luciferase activity that depends on the Rev-independent *nef *transcript was not affected by MATR3 knockdown. However, *gag *expression that is dependent on Rev-mediated export of RRE containing RNAs was greatly affected (Figure 3B). These findings suggest that MATR3 acts at a post-transcriptional level on *gag *mRNA.

**Figure 4:**
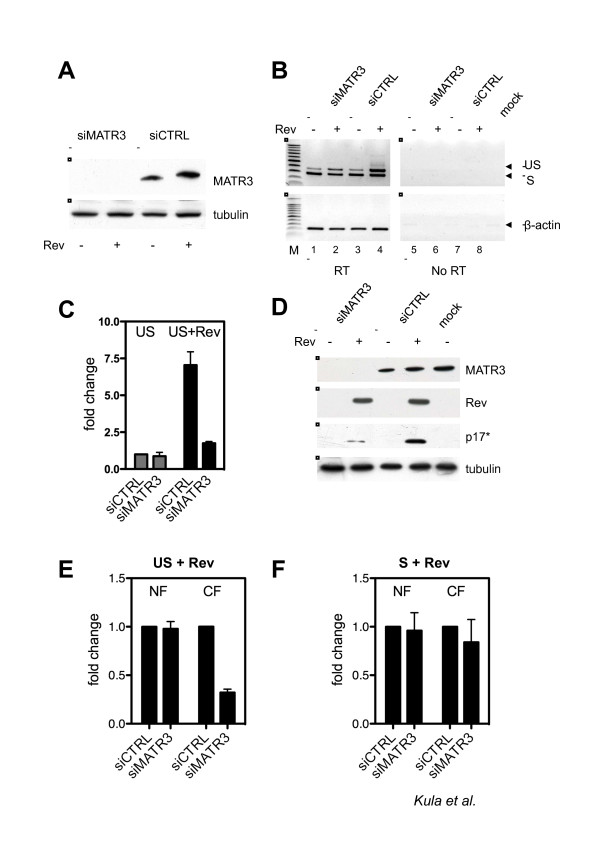
**MATR3 knockdown impairs Rev activity**. A) Knockdown of MATR3 by siRNA. 293T cells were transfected either with siRNA targeting MATR3 (siMATR3) or with a control siRNA (siCTRL) and lysed after 72 hours for western blot analysis to assess the efficiency of MATR3 knockdown. Tubulin is the protein loading control. B) RT-PCR of spliced and unspliced HIV-1 RNA levels modulated by MATR3. Spliced (S) and unspliced (US) HIV-1 RNAs were detected (lanes 1-4, upper panel) simultaneously by RT-PCR on total RNA extracted from siRNA-treated 293T cells expressing vHY-IRES-TK, Tat and Rev-EGFP as indicated. RT-PCR amplification of an unrelated RNA was not affected (β-actin mRNA) (lanes 1-4, lower panel). Reactions without RT are shown to demonstrate lack of DNA contamination (lanes 5-8). Water (mock) was used as control of DNA contamination in the reaction. C) Quantitative analysis of unspliced HIV-1 RNA levels modulated by MATR3. Unspliced (US) viral RNA expression in siRNA treated 293T cells was assayed after transfection with vHY-IRES-TK, Tat and Rev-EGFP. Unspliced RNA levels were analyzed by quantitative real-time PCR and data normalized to β-mRNA expression. Data are presented as fold change, whereby siCTRL treated cells transfected with vHY-IRES-TK and Tat in the absence of Rev were set as 1. The results of three independent experiments are shown ± SD. The inhibition was significant (p = 0.00112). D) Rev-dependent expression of HIV-1 Gag (p17*). Western blot analysis of protein extracts from siRNA-treated 293T cells expressing vHY-IRES-TK, Tat and Rev-EGFP as indicated. p17* is the product of the truncated *gag *gene of the vHY-IRES-TK vector. Tubulin is the protein loading control. E) Quantitative analysis of unspliced HIV-1 RNA levels modulated by MATR3 in the nucleus and the cytoplasm. Unspliced (US) viral RNA expression in siRNA treated 293T cells was assayed after transfection with vHY-IRES-TK, Tat and Rev-EGFP. Unspliced RNA levels were analyzed by quantitative real-time PCR on nuclear (NF) and cytoplasmic fractions (CF). Data were normalized to β-mRNA expression and presented as fold changes, whereby siCTRL 293T treated cells transfected with vHY-IRES-TK and Tat and Rev-EGFP were set as 1. The results of three independent experiments are shown ± SD. The inhibition was significant (p = 0.00091). F) Quantitative analysis of spliced HIV-1 RNA levels modulated by MATR3 in the nucleus and the cytoplasm. The experiment was conducted for spliced (S) HIV-1 RNA as described above (Figure 4E).

In order to confirm that the identified cellular factor impacts the activity of Rev, we knocked down MATR3 by siRNAs in the context of ectopic Rev expression along with Tat and the HIV-1 derived vector vHY-IRES-TK described in [57] and in Additional File 1. As shown in Figure 4A, efficient knockdown of MATR3 was obtained in the presence and absence of Rev. Next we examined the levels of unspliced viral RNA by RT-PCR. As shown in Figure 4B, in the presence of Rev, the level of unspliced viral RNA was increased due to Rev activity (compare lane 3 and 4). Interestingly, the Rev-mediated increase of unspliced HIV-1 pre-mRNA over spliced RNA was less evident when MATR3 was depleted (Figure 4B, compare lanes 1 and 2). Quantitative real-time RT-PCR (qRT-PCR) confirmed that, while depletion of MATR3 did not affect the steady-state levels of unspliced RNAs, it strongly affected its Rev-mediated increase (Figure 4C). We also demonstrated that translation of the *gag *RNA, which depends on Rev-mediated export of the corresponding RRE-containing RNA, was impaired by MATR3 knockdown (Figure 4D). To rule out any off-target effect of siRNA-mediated knockdown of MATR3 we also used a shRNA targeted to a different site. As described in Additional File 1, we observed the same phenotype on Gag expression.

**Figure 5:**
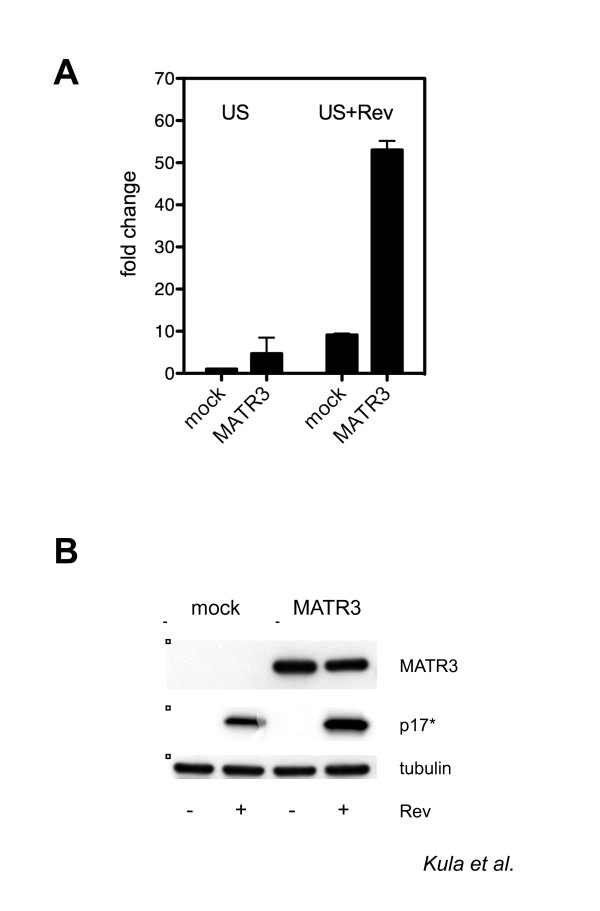
**MATR3 overexpression promotes Rev activity**. A) Quantitative analysis of unspliced HIV-1 RNA levels modulated by transfected MATR3. Unspliced (US) viral RNA expression in 293T cells was assayed after transfection with Flag-MATR3, vHY-IRES-TK, Tat and Rev-EGFP. Unspliced RNA levels were analyzed by quantitative real-time PCR and data normalized to β-mRNA expression. Data are presented as fold change, whereby 293T cells transfected with vHY-IRES-TK and Tat in the absence of Rev were set as 1. The results of three independent experiments are shown ± SD. The increase was significant (p = 0.01931). B) Transfected MATR3 upregulates Rev-dependent Gag translation. Western blot analysis of protein extracts from 293T cells expressing Flag-MATR3, vHY-IRES-TK, Tat and Rev-EGFP. p17* is the product of the truncated *gag *gene of the vHY-IRES-TKvector. Tubulin is the protein loading control

Next, we overexpressed MATR3 in cells transfected with vHY-IRES-TK, Tat, and Rev-EGFP; and we checked the levels of unspliced viral RNA by qRT-PCR. As shown in Figure 5A, Rev alone increased the amount of unspliced RNA as expected. However, overexpression of MATR3 led to a greater increase (6-folds) in the presence of Rev (Figure 5A). Consistently, translation of the *gag *RNA from the HIV-1 derived vector as shown by p17 immunoblotting was increased in the presence of transfected MATR3 (Figure 5B).

The above findings demonstrate that MATR3 impacts viral unspliced RNA and Rev-activity. However, MATR3 could act either by modulating the levels of viral RNA in the nucleus or by affecting Rev-mediated nuclear export. To address these points, we fractionated the cells and measured the levels of viral transcripts in the nucleus and in the cytoplasm. As shown in Figure 4E and 4F, the distribution of spliced RNA remained unchanged. To the contrary, only cytoplasmic Rev-dependent unspliced RNA significantly decreased when MATR3 was depleted. These results suggest that MATR3 selectively acts on the Rev-dependent nuclear to cytoplasm export of unspliced viral RNA.

### Interaction of MATR3 with Rev

**Figure 6:**
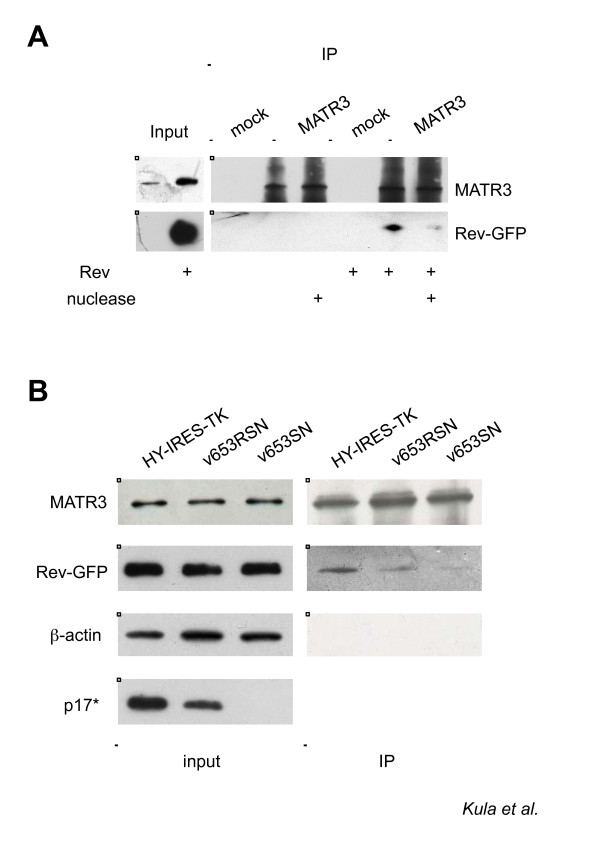
**MATR3 interaction with Rev requires HIV-1 RNA**. A) Whole cell lysates from 293T cells expressing vHY-IRES-TK and Tat with or without Rev-EGFP were subjected to immunoprecipitation with anti-MATR3 antibodies or with anti-IgG (mock). The IP were subjected to nuclease treatment and the proteins were detected by immunoblotting. B) Whole cell lysates from 293T cells expressing either vHY-IRES-TK, or v653RSN or v653SN together with Tat and Rev-EGFP were subjected to immunoprecipitation with anti-MATR3 antibodies. Immunoblots from whole cell extracts are shown on the left (input). Endogenous β-actin was used as loading control. The immunoblot for p17* shows lack of Gag expression for the RRE deficient v653SN construct (bottom panel). Immunoprecipitations are shown on the right (IP).

Finally, we sought to investigate the possible interaction between MATR3 and the Rev viral protein. To this end, we transfected 293T cells with Rev-EGFP, vHY-IRES-TK and Tat. Next, we immunoprecipitated endogenous MATR3 and found that it interacted with Rev (Figure 6A). However, the interaction appears to be RNA dependent, since the levels of Rev decreased in the presence of nuclease treatment. To discern whether or not the RRE-containing viral RNA was necessary and sufficient for the interaction with MATR3, we tested an RRE minus HIV-1 clone. To this end, we repeated the MATR3 pulldown of Rev from cells transfected either with HIV-1 vectors carrying the RRE like vHY-IRES-TK and v653RSN, the original lentiviral vector from where vHY-IRES-TK was derived [57,58]. An identical vector lacking the RRE was also used (v653SN, Additional File 1). As shown in Figure 6B, in the absence of RRE the amount of Rev that could be recovered in the pulldown was lower than in the two IPs where the RRE was present.

Taken together, our data demonstrated that MATR3, Rev and RRE-containing HIV-1 RNA are components of the same ribonucleoprotein complex.

## Discussion

Viruses are dependent on cellular partners to achieve full replication [59]. In recent years, several excellent studies have exploited unbiased screens to identify host cofactors that contribute to the HIV-1 life cycle. Genetic screens, such as transcriptome and RNAi studies [60-65], as well as interactome analysis based on yeast two-hybrid systems [66] or on proteomics [67-70] have identified essential cellular cofactors of HIV-1 infection.

In this study, we have developed a novel proteomic approach for the unbiased identification of proteins that are involved in the processing of HIV-1 RNA. The novelty of our approach relies on identifying host factors that assemble specifically on viral RNA in the context of viral transcription in the nucleus. To this end, we took advantage of the MS2 system where the RNA is tagged with binding sites for the MS2 bacteriophage coat protein [37,45]. The MS2-based method is widely used to visualize RNA by tagging the MS2 coat protein with GFP [42,43]. We exploited this system to pull down HIV-1 RNA together with associated proteins from nuclear extracts via a flag-tagged MS2 instead. Affinity purification of viral transcripts via flag-MS2, coupled to mass spectrometry, revealed several known RNA binding factors involved at various steps of cellular and/or HIV-1 RNA regulation (Table 1). Factors such as DDX3X, SFPQ, Upf1 and the Upf-1 like helicase - MOV10 have been characterized as regulators of HIV-1 RNA metabolism. DDX3X plays a role in Rev-dependent export of viral transcripts [10]. PSF, also known as splicing factor, proline- and glutamine-rich (SFPQ), binds specifically to the instability elements (INS) present in the HIV-1 genome [53]. Upf1, a key player in nonsense-mediated decay (NMD) increases stability of intron-containing HIV-1 transcripts [54]. MOV10, a putative RNA-helicase and component of P-bodies has been identified recently as a potent inhibitor of HIV-1 replication [56,71].

We focused our attention on a nuclear matrix component Matrin3 (MATR3) that co-purified with HIV-1 RNA. Knockdown of MATR3 did not affect HIV-1 transcription, but decreased Gag protein levels pointing to its involvement in a post-transcriptional step (Figure 3). The Gag protein is expressed from a subset of RRE-containing viral RNAs that are bound by the viral Rev protein and exported to the cytoplasm for gene expression. Hence, MATR3 may act as a Rev cofactor. Indeed, depletion or overexpression of MATR3 affected the total levels of unspliced viral transcripts and the amount of Gag protein (Figure 4 and 5). Interestingly, the nuclear levels of unspliced RNAs in the presence of Rev were not affected, while the cytoplasmic levels were decreased (Figure 4E). Finally we investigated the interaction of MATR3 with Rev. Our data indicate that endogenous MATR3 co-eluted with the Rev protein, but the interaction was disrupted by nuclease treatment and required the RRE element (Figure 6).

Our results are in keeping with a model where RRE-containing viral transcripts are bound by MATR3 which directs them to nuclear export in the presence of Rev. MATR3 has been characterized as a component of the nuclear matrix structure and has also been suggested to play a role in nuclear retention of hyperedited RNA with the assistance of the PSF/p54^nrb ^complex [34]. Interestingly, PSF, that is able to associate with HIV-1 RNA [53], has also been identified in our proteomic screen (Table 1), and both PSF and MATR3 have been identified in a proteomic screen of the nuclear pore [72]. We can envisage that nuclear retention and regulated Rev-mediated nuclear export of RRE-containing pre-mRNA may be regulated by these cellular factors. Alternatively, MATR3 may act in concert with the RNA helicase DDX3X (Table 1) involved in Rev/CRM1 mediated export of RRE containing transcripts [10]. Understanding of the mechanistic aspects of this process is needed to fully clarify MATR3 involvement in Rev-mediated export of viral transcripts.

## Materials and methods

### Cells and plasmids

Cells were cultivated at 37°C in Dulbecco's Modified Eagle Medium (DMEM) containing 10% FCS and antibiotics. U2OS HIV_Exo_24 × MS2 cells were obtained as described [42]. U2OS HIV_Intro_24 × MS2 cells carry the MS2 repeats in the intron and were obtained by the same protocol [19,44]. Plasmids encoding tagged versions of HIV-1 Tat and MS2 were previously described [42,73]. Plasmid MATR3-GFP was constructed by PCR amplification of the full-length cDNA (Open Biosystems cat. n. MHS1010-73974) and sub-cloning into pEGFP-N1 (Clontech). pCMV-Flag-MATR3 was obtained from Yosef Shiloh and Maayan Salton (Tel Aviv University, Israel). The HIV-1 molecular clone pNL4.3R-E-luc was kindly provided by Nathaniel Landau (New York University, USA). Plasmid Rev-EGFP was obtained from Dirk Daelemans (Rega Institute, Katholieke Universiteit Leuven, Belgium). Rev-DsRed was described in [44]. Lentiviral vectors vHY-IRES-TK, v653RSN and v653SN where described previously [57,58].

### Antibodies, western blots and immunoprecipitations

Immunoblots were performed as described before [74] with the following antibodies: MATR3 (Aviva Systems Biology, ARP40922_T100, 1:1000) or a gift from Yosef Shiloh and Maayan Salton (Tel Aviv University, Israel, 1:10000); p17 (NIH AIDS Reference Reagents Program, 1:1000); GFP (Roche, 11814460001, 1:1000); flag (Sigma, F1804, 1:1000); α-tubulin (Sigma, T5168, 1:10000); RecQL-1 (H-110) (Santa Cruz, sc-25547, 1:1000); β-actin-HRP (Sigma, A3854, 1:50000). Immunoprecipitations (IPs) were performed using the MATR3 antibody (Abcam, 70336) as described previously [74]. Briefly, 293T cells were lysed with RIPA buffer (50 mM Tris pH 7.4, 150 mM NaCl, 1% NP-40, 0.1% SDS, 1.5 mM MgCl_2_) and the cellular extracts were incubated for 4 hours with the MATR3 antibody coupled to A/G PLUS agarose beads (Santa Cruz, sc-2003) at 4°C under rotation. IPs were spun down and washed six times in RIPA buffer supplemented with 0.1 mg/ml dextran and 0.2 mg/ml heparin. Next, IPs were incubated with 40U of benzonase (Sigma, E1014) for 45 minutes at 4ºC, subsequently washed four times with RIPA buffer and eluted with 2x Laemmli buffer for SDS-PAGE.

### Preparation of nuclear extracts, RNA pull-down, mass spectrometry

To prepare nuclear extracts, U2OS cells were washed once with cold PBS and resuspended in hypotonic buffer A: 20 mM Tris HCl [pH 7.5], 10 mM NaCl, 3 mM MgCl_2_, 10% glycerol, 10 mM Ribonucleoside-Vanadyl Complex (RVC, Sigma) and the protease inhibitors cocktail (Roche). After 1 minute NP-40 was added at 0.1% v/v final concentration for 5 minutes. Nuclei were collected by low speed centrifugation at 4°C and resuspended in nuclear extraction buffer B: 20 mM Tris-HCl pH 7.5, 400 mM NaCl, 3 mM MgCl_2_, 20% glycerol additioned with RNase inhibitor RVC and the protease inhibitors cocktail as described above. After 30 minutes on ice, nuclei were subjected to three cycles of snap-freeze/thaw and insoluble proteins were removed from the nuclear extract by high-speed centrifugation at 4°C.

Nuclear extracts were adjusted to 150 mM NaCl and 0.1 mg/ml tRNA and immunoprecipitated with agarose anti-flag M2 beads (Sigma) for 3 hours at 4°C and washed eight times in wash buffer (20 mM Tris HCl pH 7.5, 300 mM NaCl, 3 mM MgCl_2_, 0.5% NP-40, 0.1 mg/ml dextran, 0.2 mg/ml heparin). Bead-bound proteins were processed for mass spectrometry analysis as described by Bish and Myers [75]. Briefly, IPs were washed for additional three times in 20 mM diammonium phosphate pH 8.0, and then incubated with 50 ng sequencing grade modified trypsin (Promega) for 8 hours at 37°C. The supernatant was removed from the beads, reduced by boiling for 5 minutes with 10 mM Tris(2-carboxyethyl)phosphine (Pierce), and alkylated with 15 mM iodoacetamide for 1 hour in the dark. An equal volume of 5% formic acid was added prior to sample cleanup with C18 ZipTips (Millipore). Samples were analyzed by LC-MS/MS using an LTQ mass spectrometer (Thermo Electron) attached to a MicroTech HPLC. LC-MS/MS data in the form of .RAW files were converted to .mzXML files by ReadW (version 1.6), and then searched against human protein databases by the Global Proteome Machine. A protein identification was considered valid when at least two non-redundant peptides from the same protein have been assigned a statistically meaningful log(e) score less than or equal to -3.0.

### RNA pulldown and RT-PCR, quantitative real-time PCR, fractionation

U2OS stable cell lines expressing Tat and flag-MS2nls were washed in cold PBS and lysed in RIPA buffer (50 mM Tris-Cl; pH 7.5, 1% NP-40, 0.05% SDS, 150 mM NaCl) plus the RNase inhibitor (Ambion) and a protease inhibitor cocktail (Roche). After 15 minutes at 4ºC the monolayer was scraped off and centrifuged at high speed. An aliquot of the resulting total extracts was saved for RNA extraction and the remaining lysates were incubated with anti-flag M2 beads (Sigma) in the presence of tRNA (0.1 mg/ml) with rotation for 3 hours. The beads were collected at 4000 rpm and were washed six times in RIPA buffer. The immunoprecipitated RNA and the total RNA were extracted using TRIzol according to the manufacturer's protocol (Invitrogen). The RNA was used as a template to synthesize cDNA using random hexamers and MMLV reverse transcriptase (Invitrogen) according to the manufacturer's protocol.

For quantitative real-time PCR, total RNA was extracted from 293T cells using TRIzol according to the manufacturer's protocol (Invitrogen).

Nuclear and cytoplasmic fractions were obtained by the following protocol. 293T cells were washed with cold PBS and resuspended in hypotonic buffer A: 20 mM Tris HCl [pH 7.5], 10 mM NaCl, 3 mM MgCl_2_, 10% glycerol and the protease inhibitors cocktail (Roche). After 1 minute NP-40 was added at 0.1% v/v final concentration for 5 minutes and cytoplasmic fraction was collected by centrifugation at 4000 rpm for 5 min. at +4°C. The pellet was washed with buffer A and the nuclei were collected by centrifugation. The cytoplasmic fraction and nuclei were subjected to RNA extraction using TRIzol according to the manufacturer's protocol (Invitrogen). Purity of fractions was assayed by Western blot of cytoplasmic and nuclear proteins.

The RNA was used as a template to synthesize cDNA using random hexamers and MMLV reverse transcriptase (Invitrogen) according to the manufacturer's protocol. Amplification of the cDNA was conducted in the presence of iQTM SYBR Green (Bio-Rad) and monitored on C1000 Thermal Cycler (Bio-Rad). Specific primers are shown in Table 2. Viral RNA abundance is normalized to β-actin mRNA expression and shown as fold change in comparison with control samples. Results were expressed as mean plus or minus SD. Significant expression changes are represented by P < 0.05. The two-tailed student-T test confirmed significant expression changes in the results.

**Table 2:** Primers for RT-PCR

Name	Sequence 5' > 3'
A (nuc1b-177)	CGAGATCCGTTCACTAATCGAATG

B	GGATTAACTGCGAATCGTTCTAGC

C	CGAGATCCGTTCACTAATCGAATG

BA1 (β-actin)	CATGTGCAAGGCCGGCTTCG

BA4 (β-actin)	GAAGGTGTGGTGCCAGATTT

### siRNA- mediated knockdown of MATR3

Pools of siRNAs were obtained from Dharmacon: MATR3 siGENOME SmartPool (UAGAUGAACUGAGUCGUUA, GACCAGGCCAGUAACAUUU, ACCCAGUGCUUGAUUAUGA, CCAGUGAGAGUUCAUUUAU), siGENOME Non-Targeting siRNA Pool #1. Either HeLa cells or 293T cells were transfected with siRNAs at the concentration of 100 nM and with HiPerFect Transfection Reagent (Qiagen) according to manufacturer's instructions and previous protocols [76]. After 48 hours the efficiency of the knockdown was analyzed at the protein level by Western blot.

A short-hairpin shRNA targeted to MATR3 (Open Biosystems individual clone ID: TRCN0000074905) delivered by a lentiviral vector (pLKO.1), or a control targeting luciferase (courtesy of Dr. Ramiro Mendoza-Maldonado), were produced in 293T cells by cotransfection with the packaging plasmids psPAX2 and pMD2.G using 5 μg/ml polybrene. Supernatants were used to transduce 293T cells. After 48 hours cells were assayed for MATR3 expression and transfected.

### Luciferase assay

HeLa cells, treated with siRNA as described above, were transfected with the pNL4.3R-E-luc HIV-1 molecular clone along with the pCMV-Renilla vector. Twenty-four hours after transfection, the cells were harvested and lysed in passive lysis buffer (Promega) and the levels of luciferase activity were measured by the Dual-Luciferase-Reporter assay (Promega) as directed by manufacturers. For normalization, total protein concentration in each extract was determined with a Bio-Rad protein assay kit.

## Competing interests

The authors declare that they have no competing interests.

## Authors' contributions

AK performed all the experiments and analyzed the data. JG participated in the real-time PCR. AK participated in the characterization of the cell clones. DK participated in the knockdown experiments. MPM produced and analyzed the proteomic data. AM conceived and coordinated the study, analyzed the data and wrote the manuscript. All authors read and approved the final manuscript.

## Supplementary Material

Additional file 1 **Supplementary information on the characterization of the vectors, on their Rev-responsiveness and on shRNA-mediated knockdown of MATR3**. Click here for file
