# Supplementary material for: Characterization of the HIV-1 RNA associated proteome identifies Matrin 3 as a nuclear cofactor of Rev function
Source: Retrovirology. 2011 Jul 20;8:60. doi: 10.1186/1742-4690-8-60 (PMC3160904; doi:10.1186/1742-4690-8-60)
Supplement: Additional file 1 — Supplementary information on the characterization of the vectors, on their Rev-responsiveness and on shRNA-mediated knockdown of MATR3. [file 1742-4690-8-60-S1.PDF]

Additional File 1

Supplementary information for 'Characterization of the HIV-1 RNA associated proteome identifies Matrin 3 as a nuclear cofactor of Rev function' by Kula, A. et al.

Characterization of the cell clones for MS2-tagging of viral RNA.

The MS2-tagging method has been used to study transcription in a variety of cell systems, including bacteria, yeast and mammalian cells [1] [2-5]. The method of MS2 tagging of RNA has been applied to HIV-1 to study transcription [6-9] and Gag assembly [10-12]. Therefore, MS2 tagging of RNA is a solid and extensively used approach to study HIV RNA transcription dynamics.

The HIVexo transcript has been extensively characterized in a previous publication [6]. Integrity of the transcript and the various processed forms was demonstrated by quantitative *in situ* RNA hybridization (RNA Q-ISH). Recruitment of factors like RNAPII, Cyclin T1 and CDK9 at the transcription site confirmed active transcription.

Also the HIV-Intro transcript has been extensively characterized [13]. This construct has the advantage of carrying a reporter for gene expression (ECFPskl) that is well expressed in the presence of MS2 (see western blot in Figure 1B, lane 5 and 7). Further analysis included: (i) RT-PCR of the HIV-Intro transcript with or without MS2 and in the presence of Rev; (ii) ECFP signal in the cytoplasmic peroxisomes in the presence of EYFP-MS2nls; (iii) Recruitment of RNAPII and Cyclin T1 at the transcription site [13].

Finally, a manuscript describing in great detail the kinetic behavior of HIV-1 transcription exploiting the HIV-Intro construct has been submitted elsewhere (Maiuri et al. 2011, submitted).

Additional characterization of the U20S HIVintro cell line is presented in Additional File 2.

Additional File 2

Characterization of the U20S HIVintro cell line.

A) U2OS HIVintro cells expressing EYFP-MS2nls were transfected with Tat-cherry. The transcription site marked by EYFP-MS2nls staining is indicated by an arrow and is enriched with Tat-cherry. ECFPskl spotted signal in the cytoplasm is an indication of correct gene expression [14]. Above, diagram of the HIVintro construct showing the position of the primers for RT-PCR as in Figure 1 and the position of the probes for RNA ISH (red). The sequences of the ISH probes where described previously [6].

B) *In situ* hybridization of HIV-1 RNA. The probe hybridizing in the intron (left) shows a clear spot of transcribed RNA in the nucleus (arrow) and no cytoplasmic staining. The probe hybridizing in the exon (right) shows both staining at the transcription site (arrow) and in the cytoplasm.

C) Quantitative *in situ* hybridization of HIV-1 RNA at the site of transcription. Fold changes of probe E1 over the 3'-end probe (E2) are shown (N = 20 ± standard deviation). Labelled probes were described previously in detail [6] and their position is indicated in A.

D) RT-PCR of HIV-1 RNA. The assay was performed on U2OS HIVintro cells as described in [14] and shows the lack of major effects of expressing EYFP-MS2nls on HIV-1 transcription and splicing. RT PCR was performed with the primers A, B and C shown in panel A.

Rev-responsiveness of the vectors.

All the vectors used in this study are Rev-responsive. vHY-IRES-TK encodes for a truncated Gag and its expression leads to the p17* protein when Rev-EGFP or Rev-DsRed was expressed (Additional File 3). The HIV vectors carrying the MS2 tag do not express Gag (see below), but their responsiveness to Rev was assessed looking at the unspliced viral RNA in the cytoplasm (Additional File 3B&C). EYFP-MS2nls was detectable in the cytoplasm only in the presence of Rev-DsRed.

Additional File 3

Responsiveness of MS2-tagged HIV vectors to Rev activity.

A) Rev-dependent expression of HIV-1 Gag (p17*). Western blot analysis of protein extracts from 293T cells expressing vHY-IRES-TK, Tat and either Rev-EGFP or Rev-DsRed as indicated. p17* is the product of the truncated gag gene of the vHY-IRES-TK vector. Tubulin is the protein loading control.

B&C) Export of unspliced HIV-1 RNA in the presence of Rev. U2OS HIVintro cells expressing EYFP-MS2nls and Tat were transfected either with Rev-mDsRed (A) or with the mDsRed control (B). Rev-mDsRed mediates the nuclear export of unspliced HIV-1 RNA, which is visualized by EYFP-MS2nls in the cytoplasm. MS2 staining in the nucleus is shown in yellow with the transcription site indicated by an arrow. Insets shown below figures represent enlargements of the indicated cytoplasmic regions acquired with the same settings in A and B to highlight the presence of HIV-1 RNA in the cytoplasm, avoiding the strong EYFP-MS2nls signal coming from the nucleus.

Description of the HIV-1 vectors used to characterize MATR3

At the early days of lentiviral vectors the Sodroski laboratory produced a series of vectors [15]. The best performing v653RSN maintained a portion of the gag gene with a functional ATG that allowed the expression of a truncated form of Gag (p17*). vHY-IRES-TK was derived from that vector [16]. However, the most popular series of lentiviral vectors was produced by Naldini/Trono [17]. These vectors were almost identical to those of Parolin/Sodroski but carry a mutation of the ATG of gag that precludes the translation of the mRNA. The MS2-tagged HIV intro and HIVexo were derived from these constructs. Therefore, in order to be able to detect Gag, we used the HIV-HY-IRES-TK and v653RSN. A more detailed description is shown in Additional File 4.

Additional File 4

Schematic view of the lentiviral vectors used in this study.

A) The vHY-IRES-TK vector was described in [16]. Briefly, the vector was derived from v653RSN (see below) by inserting a cassette expressing hygromycin resistance gene (HY) and the HSV-1 thymidine kinase gene (TK) separated by an internal ribosome entry site (IRES).

B) The v653RSN vector was described in [15]. Briefly, the vector carries a portion of gag for efficient packaging and a portion of env encompassing the Rev-responsive element (RRE). The SL3 Neo transcription unit was used for clone selection.

C) The v653SN vector is identical to the v653RSN vector but lacks the RRE. This vector was described in [15].

Additional File 5

shRNA-mediated knockdown of MATR3.

In order to confirm the results obtained with the knockdown of MATR3 exploiting the MATR3 siGENOME SmartPool from Dharmacon we performed an experiment where the knockdown was obtained with a short-hairpin RNA (shRNA). 293T cells transduced with a lentiviral vector encoding the MATR3 shRNA (pLKO.1 from Open Biosystems individual clone ID: TRCN0000074905), or a control targeting luciferase (courtesy of Dr. Ramiro Mendoza-Maldonado), where then transfected with vHY-IRES-TK, Tat and Rev. Efficiency of knockdown and expression of p17* from vHY-IRES-TK was monitored by Western blot analysis.

References

1. Bertrand E, Chartrand P, Schaefer M, Shenoy SM, Singer RH, Long RM: **Localization of ASH1 mRNA particles in living yeast.** *Mol Cell* 1998, **2:**437-445.
2. Golding I, Paulsson J, Zawilski SM, Cox EC: **Real-time kinetics of gene activity in individual bacteria.** *Cell* 2005, **123:**1025-1036.
3. Janicki SM, Tsukamoto T, Salghetti SE, Tansey WP, Sachidanandam R, Prasanth KV, Ried T, Shav-Tal Y, Bertrand E, Singer RH, Spector DL: **From**

silencing to gene expression: real-time analysis in single cells. *Cell* 2004, **116:**683-698.

4. Shav-Tal Y, Darzacq X, Shenoy SM, Fusco D, Janicki SM, Spector DL, Singer RH: **Dynamics of single mRNPs in nuclei of living cells.** *Science* 2004, **304:**1797-1800.
5. Karpova TS, Kim MJ, Spriet C, Nalley K, Stasevich TJ, Kherrouche Z, Heliot L, McNally JG: **Concurrent fast and slow cycling of a transcriptional activator at an endogenous promoter.** *Science* 2008, **319:**466-469.
6. Boireau S, Maiuri P, Basyuk E, de la Mata M, Knezevich A, Pradet-Balade B, Backer V, Kornblihtt A, Marcello A, Bertrand E: **The transcriptional cycle of HIV-1 in real-time and live cells.** *J Cell Biol* 2007, **179:**291-304.
7. Molle D, Maiuri P, Boireau S, Bertrand E, Knezevich A, Marcello A, Basyuk E: **A real-time view of the TAR:Tat:P-TEFb complex at HIV-1 transcription sites.** *Retrovirology* 2007, **4:**36.
8. du Chene I, Basyuk E, Lin YL, Triboulet R, Knezevich A, Chable-Bessia C, Mettling C, Baillat V, Reynes J, Corbeau P, et al: **Suv39H1 and HP1gamma are responsible for chromatin-mediated HIV-1 transcriptional silencing and post-integration latency.** *Embo J* 2007, **26:**424-435.
9. Maiuri P, Knezevich A, Bertrand E, Marcello A: **Real-time imaging of the HIV-1 transcription cycle in single living cells.** *Methods* 2010.
10. Camus G, Segura-Morales C, Molle D, Lopez-Verges S, Begon-Pescia C, Cazevieille C, Schu P, Bertrand E, Berlioz-Torrent C, Basyuk E: **The clathrin adaptor complex AP-1 binds HIV-1 and MLV Gag and facilitates their budding.** *Mol Biol Cell* 2007, **18:**3193-3203.
11. Jouvenet N, Simon SM, Bieniasz PD: **Imaging the interaction of HIV-1 genomes and Gag during assembly of individual viral particles.** *Proc Natl Acad Sci U S A* 2009, **106:**19114-19119.
12. Molle D, Segura-Morales C, Camus G, Berlioz-Torrent C, Kjems J, Basyuk E, Bertrand E: **Endosomal trafficking of HIV-1 gag and genomic RNAs regulates viral egress.** *J Biol Chem* 2009, **284:**19727-19743.
13. De Marco A, Biancotto C, Knezevich A, Maiuri P, Vardabasso C, Marcello A: **Intragenic transcriptional cis-activation of the human immunodeficiency virus 1 does not result in allele-specific inhibition of the endogenous gene.** *Retrovirology* 2008, **5:**98.
14. De Marco A, Dans PD, Knezevich A, Maiuri P, Pantano S, Marcello A: **Subcellular localization of the interaction between the human immunodeficiency virus transactivator Tat and the nucleosome assembly protein 1.** *Amino Acids* 2009.
15. Parolin C, Dorfman T, Palu G, Gottlinger H, Sodroski J: **Analysis in human immunodeficiency virus type 1 vectors of cis-acting sequences that affect gene transfer into human lymphocytes.** *J Virol* 1994, **68:**3888-3895.
16. Marcello A, Giaretta I: **Inducible expression of herpes simplex virus thymidine kinase from a bicistronic HIV1 vector.** *Res Virol* 1998, **149:**419-431.
17. Naldini L, Blomer U, Gallay P, Ory D, Mulligan R, Gage FH, Verma IM, Trono D: **In vivo gene delivery and stable transduction of nondividing cells by a lentiviral vector.** *Science* 1996, **272:**263-267.

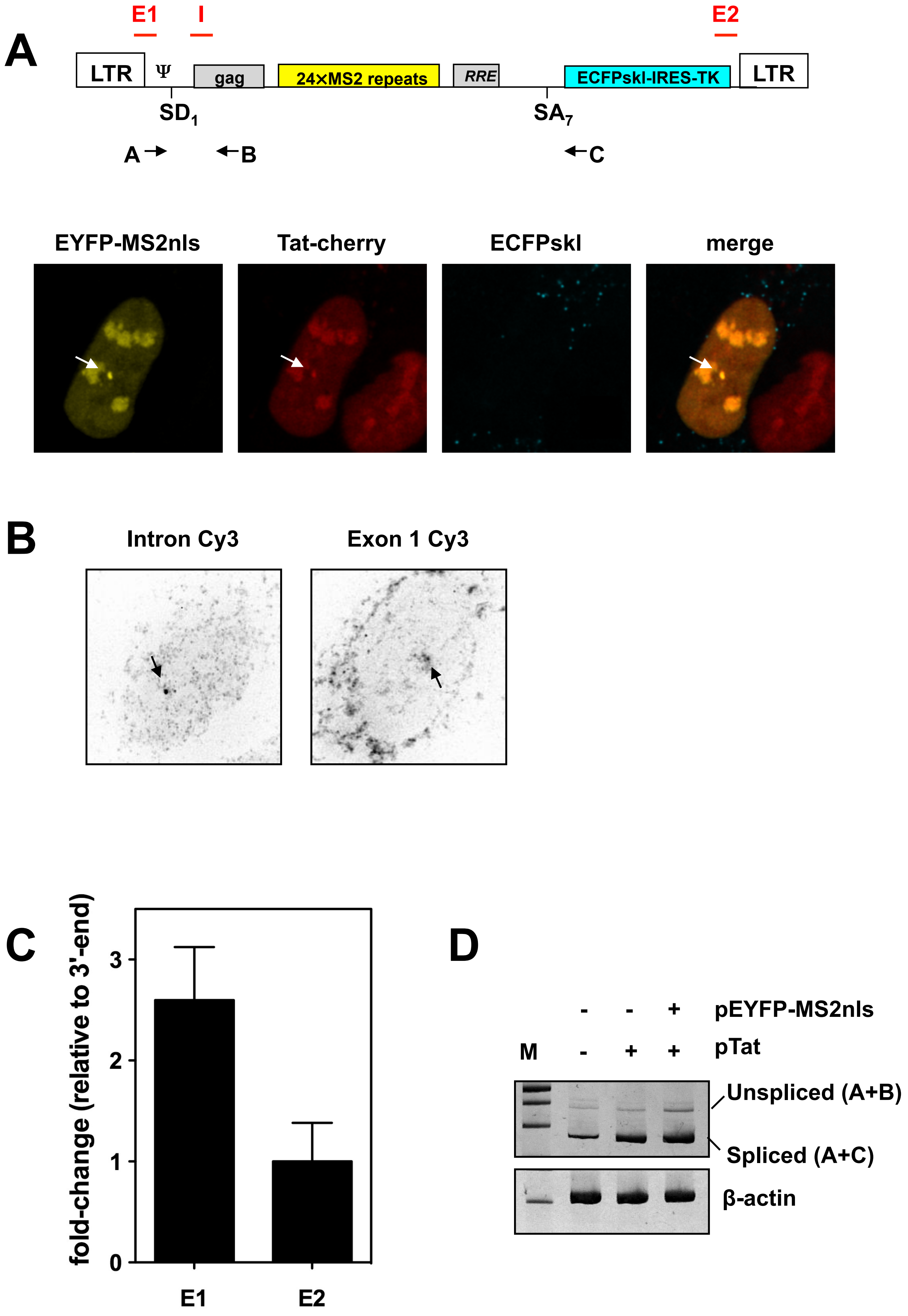

Additional File 2 - Kula et al.

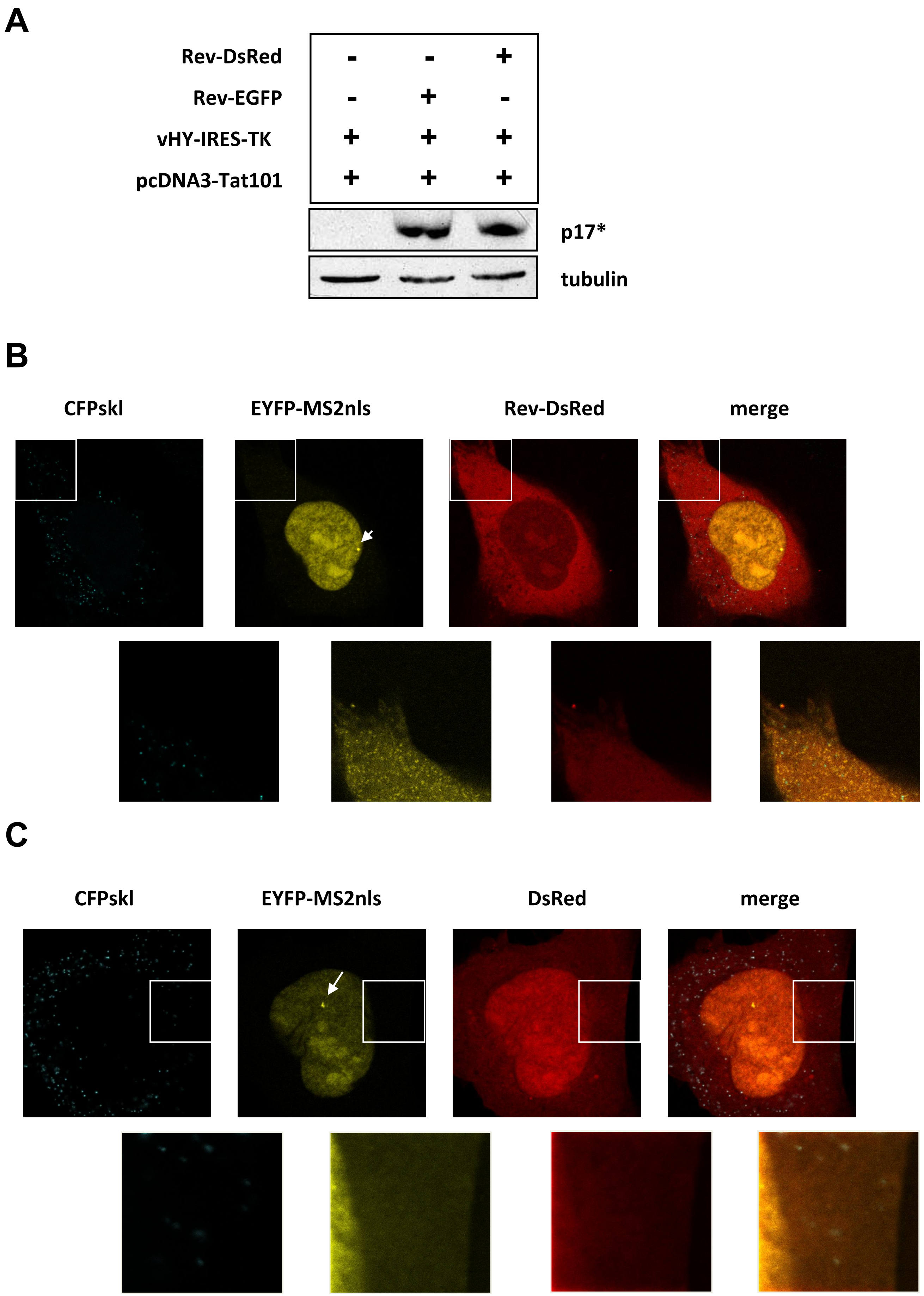

Additional File 3 - Kula et al.

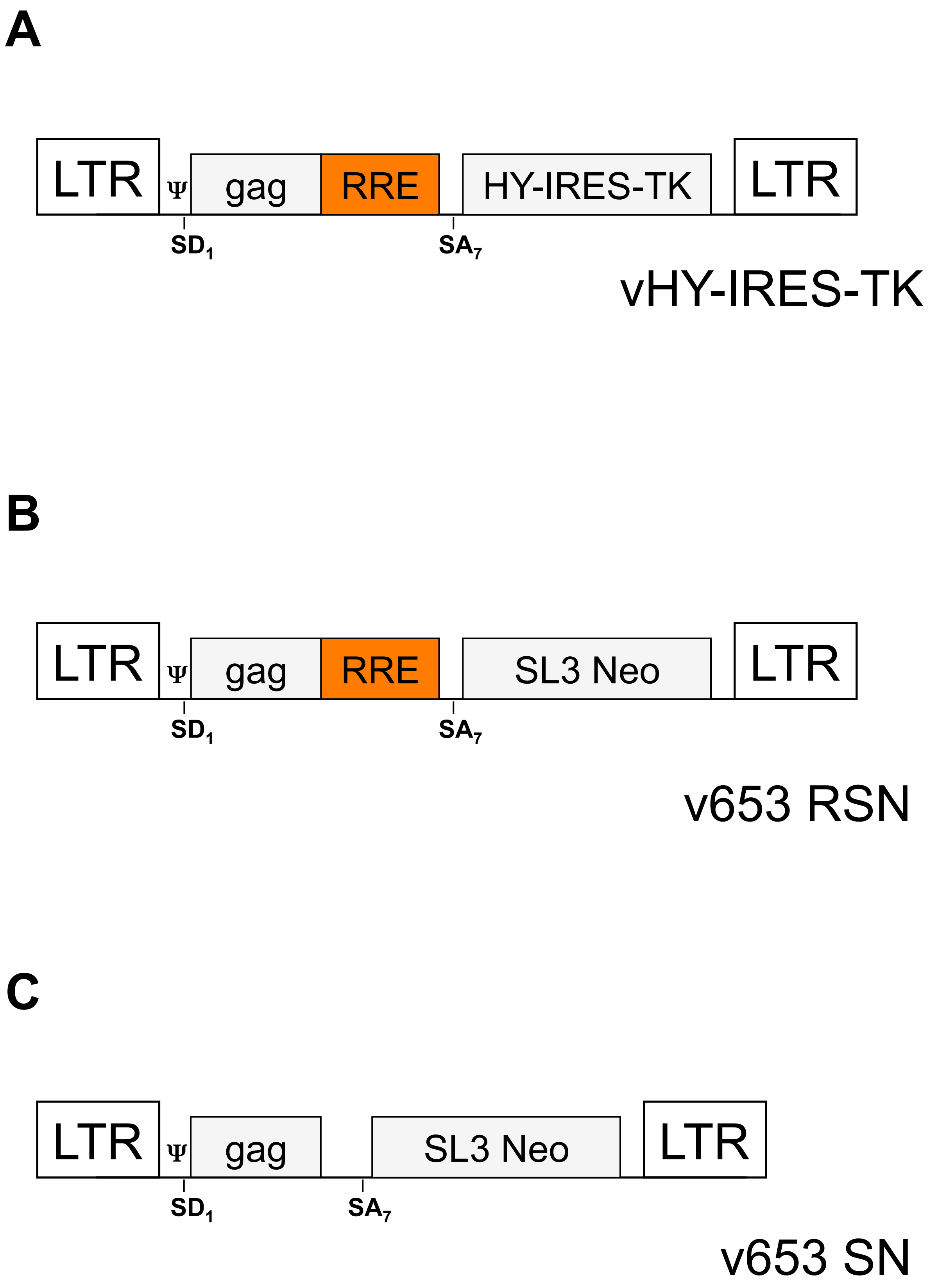

Additional File 4 - Kula et al.

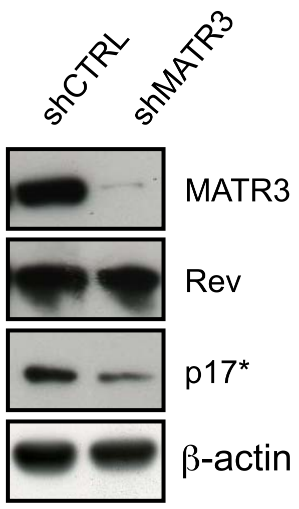

Additional File 5 - Kula et al.
